# Evaluation of a smartphone human activity recognition application with able-bodied and stroke participants

**DOI:** 10.1186/s12984-016-0114-0

**Published:** 2016-01-20

**Authors:** N. A. Capela, E. D. Lemaire, N. Baddour, M. Rudolf, N. Goljar, H Burger

**Affiliations:** Ottawa Hospital Research Institute, Ottawa, Canada; Faculty of Medicine, University of Ottawa, Ottawa, Canada; Mechanical Engineering, University of Ottawa, Ottawa, Canada; University Rehabilitation Institute, Ljubljana, Slovenia

**Keywords:** Activities of Daily Living, Monitoring, Ambulatory/instrumentation, Cellular Phone, Movement, Accelerometry/instrumentation

## Abstract

**Background:**

Mobile health monitoring using wearable sensors is a growing area of interest. As the world’s population ages and locomotor capabilities decrease, the ability to report on a person’s mobility activities outside a hospital setting becomes a valuable tool for clinical decision-making and evaluating healthcare interventions. Smartphones are omnipresent in society and offer convenient and suitable sensors for mobility monitoring applications. To enhance our understanding of human activity recognition (HAR) system performance for able-bodied and populations with gait deviations, this research evaluated a custom smartphone-based HAR classifier on fifteen able-bodied participants and fifteen participants who suffered a stroke.

**Methods:**

Participants performed a consecutive series of mobility tasks and daily living activities while wearing a BlackBerry Z10 smartphone on their waist to collect accelerometer and gyroscope data. Five features were derived from the sensor data and used to classify participant activities (decision tree). Sensitivity, specificity and F-scores were calculated to evaluate HAR classifier performance.

**Results:**

The classifier performed well for both populations when differentiating mobile from immobile states (F-score > 94 %). As activity recognition complexity increased, HAR system sensitivity and specificity decreased for the stroke population, particularly when using information derived from participant posture to make classification decisions.

**Conclusions:**

Human activity recognition using a smartphone based system can be accomplished for both able-bodied and stroke populations; however, an increase in activity classification complexity leads to a decrease in HAR performance with a stroke population. The study results can be used to guide smartphone HAR system development for populations with differing movement characteristics.

## Background

Mobile health monitoring using wearable sensors is a growing area of interest. As the world’s population ages and locomotor capabilities decrease, the ability to monitor a person’s mobility activities outside a hospital setting becomes valuable for clinical decision-making. Human Activity Recognition (HAR) systems combine wearable sensor and computing technologies to monitor human movement in the person’s chosen environment.

HAR systems typically use accelerometer and gyroscope sensors since these are small, affordable, and generally unobtrusive [1]. Other HAR systems combine sensor types, such as accelerometer and ECG [2], or use multiple sensor locations, such as sternum and thigh [3], or thigh and chest [4]. However, multiple sensors can be cumbersome and inconvenient for reliable implementation in everyday life. Smartphones are ubiquitous, carried by most individuals on a daily basis, and many devices contain integrated accelerometer and gyroscope sensors, which are commonly used to measure posture and movement [5].

HAR systems typically follow a machine learning structure [6]. Raw sensor signals are collected, pre-processed, and segmented into time windows. Feature extraction is then performed to retrieve relevant information from sensor signals over each window. Features are abstractions of raw data; such as statistical calculations (mean, variance etc.) or frequency domain features that describe the signal’s periodic structure. Since many features could be used in a model, a selection process is typically used to reduce the data’s dimensionality. Feature selection methods may be filter-based, which evaluate features characteristics without a classifier, or wrapper based, which use classifier accuracy to evaluate features [7]. Finally, a classifier is constructed using training data and evaluated on testing data. The literature has previously focused on offline human activity recognition, although recent work is moving towards algorithms that can be implemented in real time using the onboard sensors and computational power of a smartphone [8].

Many HAR systems have been developed for able-bodied participants; however, few systems have been tested on the elderly or people with disabilities [9]. A recent study showed that an activity classification model trained on an older cohort and tested on a younger sample performed better than model training with the younger cohort and testing on the older sample. This suggested that a model trained on elderly participants may be more generalizable and result in more a robust classifier [10], since younger people may perform activities of daily living with more intensity than older or disabled people. Stroke is a leading cause of disability among adults and can lead to limited activities of daily living, balance and walking problems, and a need for constant care [11]. For a clinician, reliable data about a patient’s activity is important, particularly information about the type, duration and frequency of daily activities (i.e., standing, sitting, lying, walking, climbing stairs). This information can help therapists design rehabilitation programmes and monitor progress of patients outside of the hospital. An objective record of a patient’s daily activities can avoid mistaken or intentionally misleading self-reporting. Mobility monitoring could provide large datasets with information about the mobility habits of people who have suffered a stroke, guiding future research in the field of healthcare and intervention.

The current research compared the performance of a smartphone-based wearable mobility monitoring system (WMMS) between able-bodied participants and people who had suffered a stroke. By studying differences in classifier performance between populations, we addressed the hypothesis that a WMMS developed using sensor data from able-bodied participants would perform worse on a population of stroke participants due to differences in walking biomechanics. This research also identified where the classifier performed poorly, thereby providing guidance for future research on HAR for populations with mobility problems.

## Methods

### Population

A convenience sample of 15 able-bodied participants (age 26 ± 8.9 years, height 173.9 ± 11.4 cm, weight 68.9 ± 11.1 kg) and 15 stroke participants (age 55 ± 10.8 years, height 171.6 ± 5.79 cm, weight 80.7 ± 9.65 kg) participated in this study. Stroke participants were recruited at the University Rehabilitation Institute in Ljubljana, Slovenia, and able-bodied participants were recruited at the Ottawa Hospital Rehabilitation Centre in Ottawa, Canada. Stroke participants were identified by a physical and rehabilitation medicine specialist as capable of safely completing the mobility tasks and able to commit to the time required to complete the evaluation session (approximately 30 min). Six stroke patients had left hemiparesis and nine had right hemiparesis. Thirteen stroke patients had ischemic stroke, one subarachnoid hemorrhage and one had impairment because of a benign cerebral tumor. Six stroke patients used one crutch, two had one arm in a sling, and one used an ankle-foot orthosis. The stroke event averaged 9.6 months before the study and the average FIM score was 107 points. The study was approved by the Ottawa Health Science Network Research Ethics Board and the Ethics Board of University Rehabilitation Institute (Ljubljana, Slovenia). All participants provided informed consent.

### Equipment

Accelerometer, magnetometer, and gyroscope data were collected with a Blackberry Z10 smartphone using the TOHRC Data Logger [12] in both the Ottawa and Ljubljana locations. Smartphone sampling rates can vary [13], therefore the Z10 sensors were sampled at approximately 50Hz, with a mean standard deviation of 15.37Hz across all trials. The WMMS used the Blackberry’s gravity and linear acceleration output to calculate features. Linear acceleration is the Z10 acceleration minus the acceleration due to gravity. On the BlackBerry Z10, the inertial measurement unit fuses the accelerometer, gyroscope, and magnetometer sensors and splits acceleration components into applied linear acceleration and acceleration due to gravity (the gravity signal); however, the device manufacturer does not report how this is accomplished.

Since the phone’s orientation on the pelvis can differ between individuals due to a larger mid-section or different clothing, a rotation matrix method was used to correct for phone orientation [14]. Ten seconds of accelerometer data were collected while the participant was standing still and a 1-s data segment with the smallest standard deviation was used to calculate the rotation matrix constants. The orientation correction matrix was applied to all sensor data.

While the WMMS application can run entirely on the smartphone, for the purposes of this research, the raw sensor output was exported as a text file and run in a custom Matlab program to observe WMMS algorithm performance in detail and calculate outcome measures.

### WMMS algorithm

Raw sensor data from the smartphone were converted into features, over 1 s data windows. Data interpolation was not used and, since the results remained acceptable, this method was not sensitive to within window sampling rate variability with a standard deviation of 15.37 Hz.”. The features were used to classify movement activities. The features derived from acceleration due to gravity, linear acceleration, and gyroscope signals are displayed in Table 1. Features were selected based on the literature and observing feature behaviour from pilot data with the target activities.

**Table 1:** Features derived from smartphone sensor signals. Acceleration due to gravity = (Xgrav, Ygrav, Zgrav), linear acceleration = (Xlin, Ylin, Zlin), SD = standard deviation

Signal Feature	Formula	Abbreviation
Simple moving average of sum of range of linear acceleration (4 windows)	$\frac{{\varSigma^4}_{\mathrm{i}=1}\left[\left( range\left(Xli{n}_i\right)\right) \pm \left( range\left(Yli{n}_i\right)\right) \pm \left( range\left(Zli{n}_i\right)\right)\right]}{4}$	L-SMA
Difference to Y	*Ygrav*–*Zgrav*–*Xgrav*	DifftoY
Sum of range of linear acceleration	(*range*(*Xlin*_*i*_)) + (*range*(*Ylin*_*i*_)) + (*range*(*Zlin*_*i*_))	SOR
Sum of standard deviation of linear acceleration	(*SD*(*Xlin*_*i*_)) + (*SD*(*Ylin*_*i*_)) + (*SD*(*Zlin*_*i*_))	SoSD
Maximum slope of simple moving average of sum of variances of gravity	$\frac{SM{A}_{var} = {\varSigma^4}_{\mathrm{i}=1}\left(Var{(Xgrav)}_i \pm Var{(Ygrav)}_i \pm Var{(Zgrav)}_i\right)}{4}$ *max*(*SMA*_*var*_(2)–*SMA*_*var*_(1), *SMA*_*var*_(3)–*SMA*_*var*_(2), *SMA*_*var*_(4)–*SMA*_*var*_(3))	G-SMAvar

**Fig. 1:**
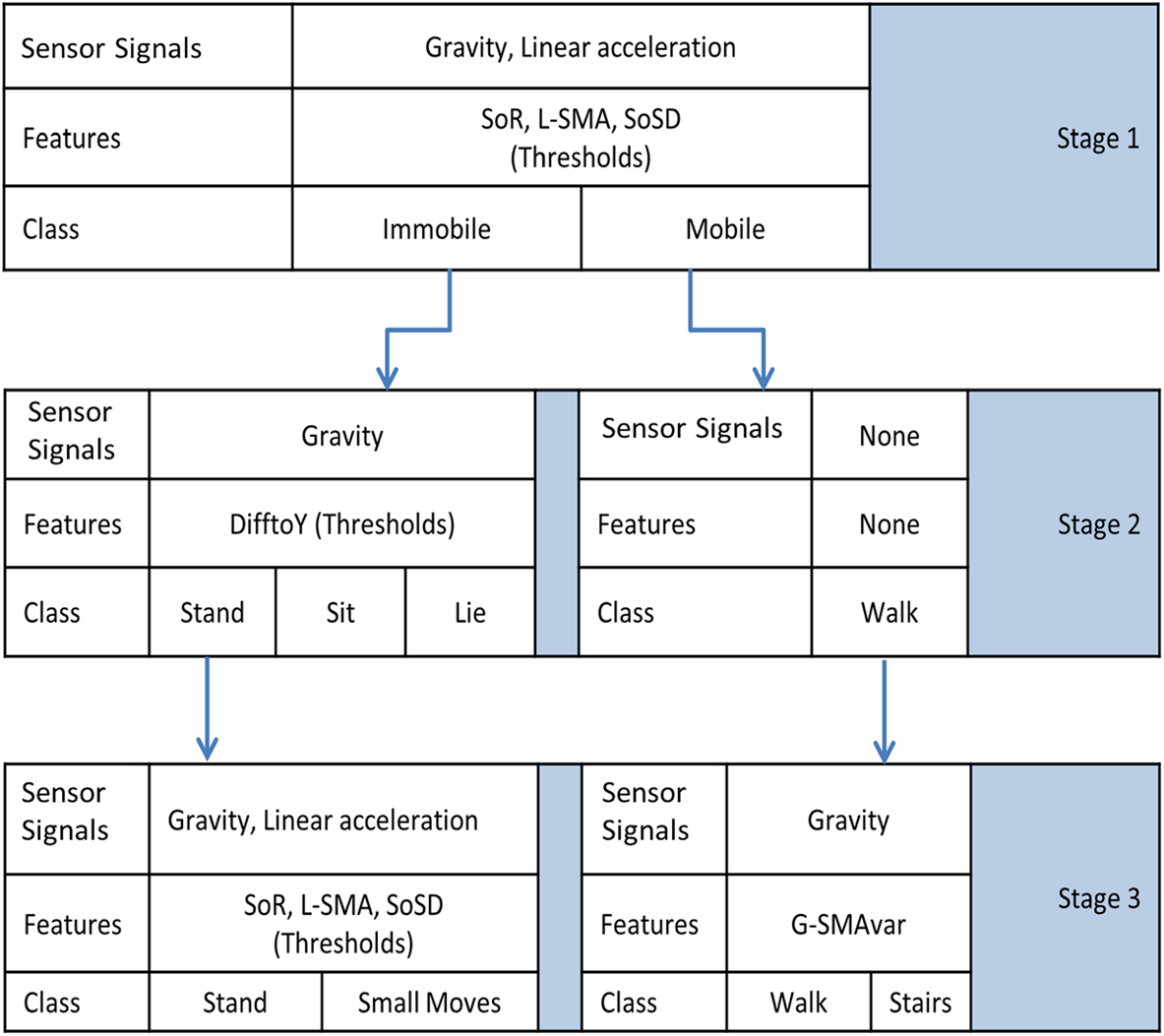
WMMS Decision Tree Structure

A custom decision tree used these features to classify six activity states: mobile (walk, stairs) and immobile (sit, stand, lie, and small movements). The decision tree structure is shown in Fig. 1.

**Fig. 2:**
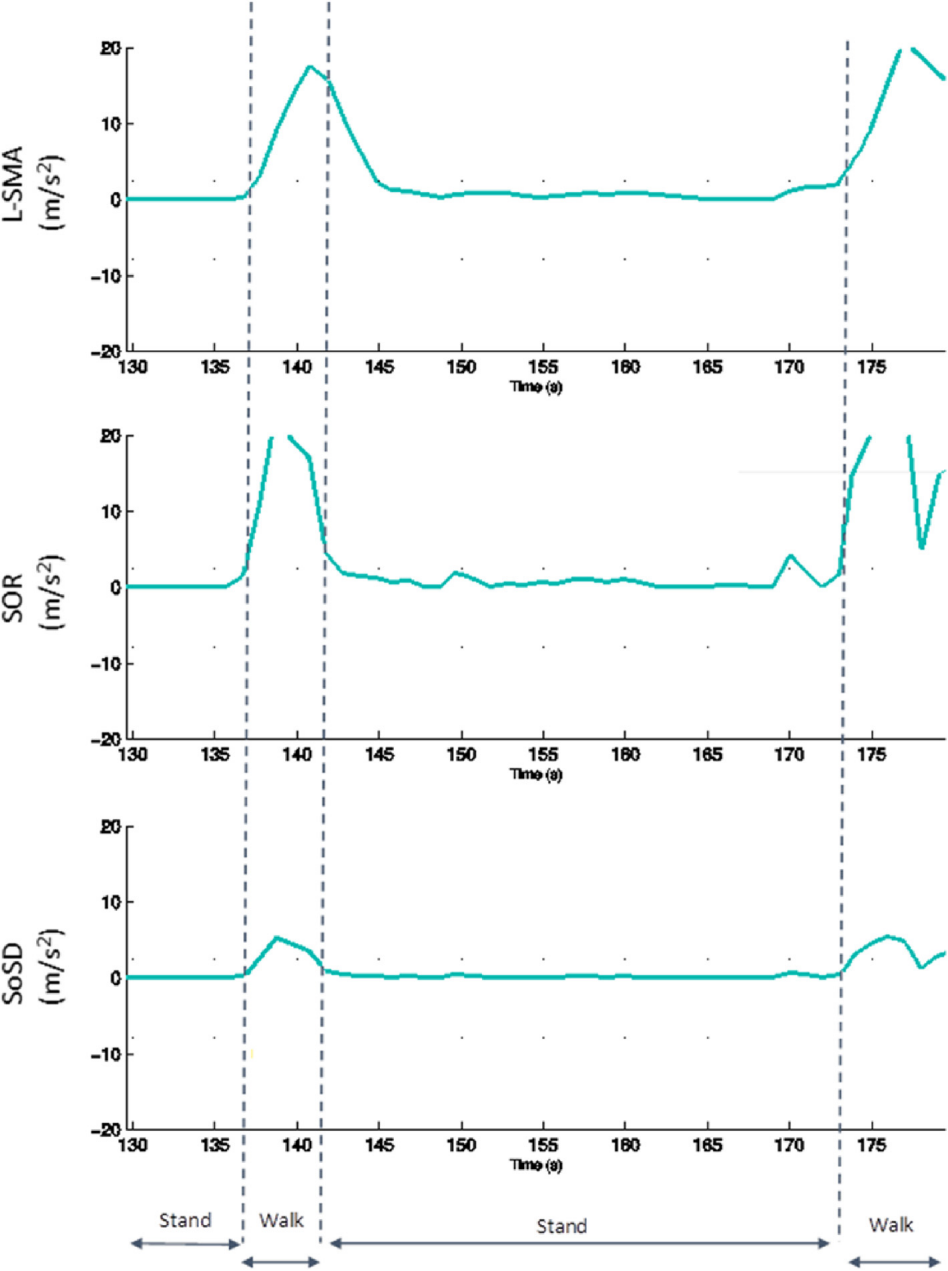
Plots of L-SMA, SOR and SoSD showing how these features change during mobile and immobile activities

The WMMS has three activity stages. The first stage used a combination of three features (L-SMA, SOR, SoSD: Table 1) to identify if the person was mobile (walking, climbing stairs) or immobile (sitting, standing, lying down, or small movements). All thresholds were determined using a separate experimental set of able-bodied participant data, collected for this purpose. Figure 2 shows plots of L-SMA, SOR, and SoSD that demonstrate how these features change during immobile and mobile activities.

**Fig. 3:**
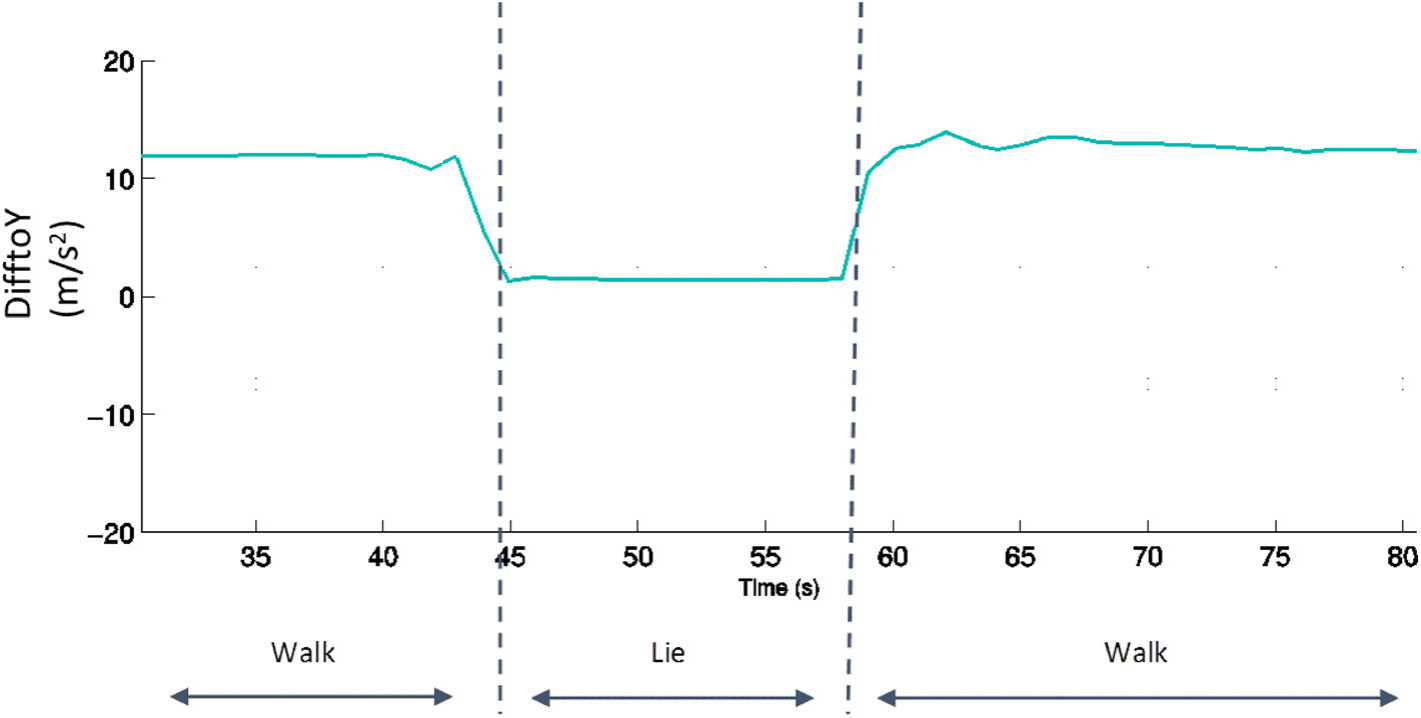
Plot of DifftoY showing how this feature change during waling and lying down activities

In stage 2, if the person was in an immobile state, trunk orientation was examined using the “difference to Y” signal feature (Table 1). Based on thresholds, the classifier determined if the person was upright (standing), leaning back (sitting), or horizontal (lying down). If the person was standing, a weighting factor was calculated based on how many of the stage 1 features passed thresholds. If the weighting factor exceeded 1 for two consecutive data windows and the person was standing for more than 3 s, the person was considered to be performing a small movement (i.e., standing and washing dishes at a sink, etc.). Figure 3 shows how the DifftoY feature changes when a person walks to a bed, lies down, and stands up again to continue walking.

**Fig. 4:**
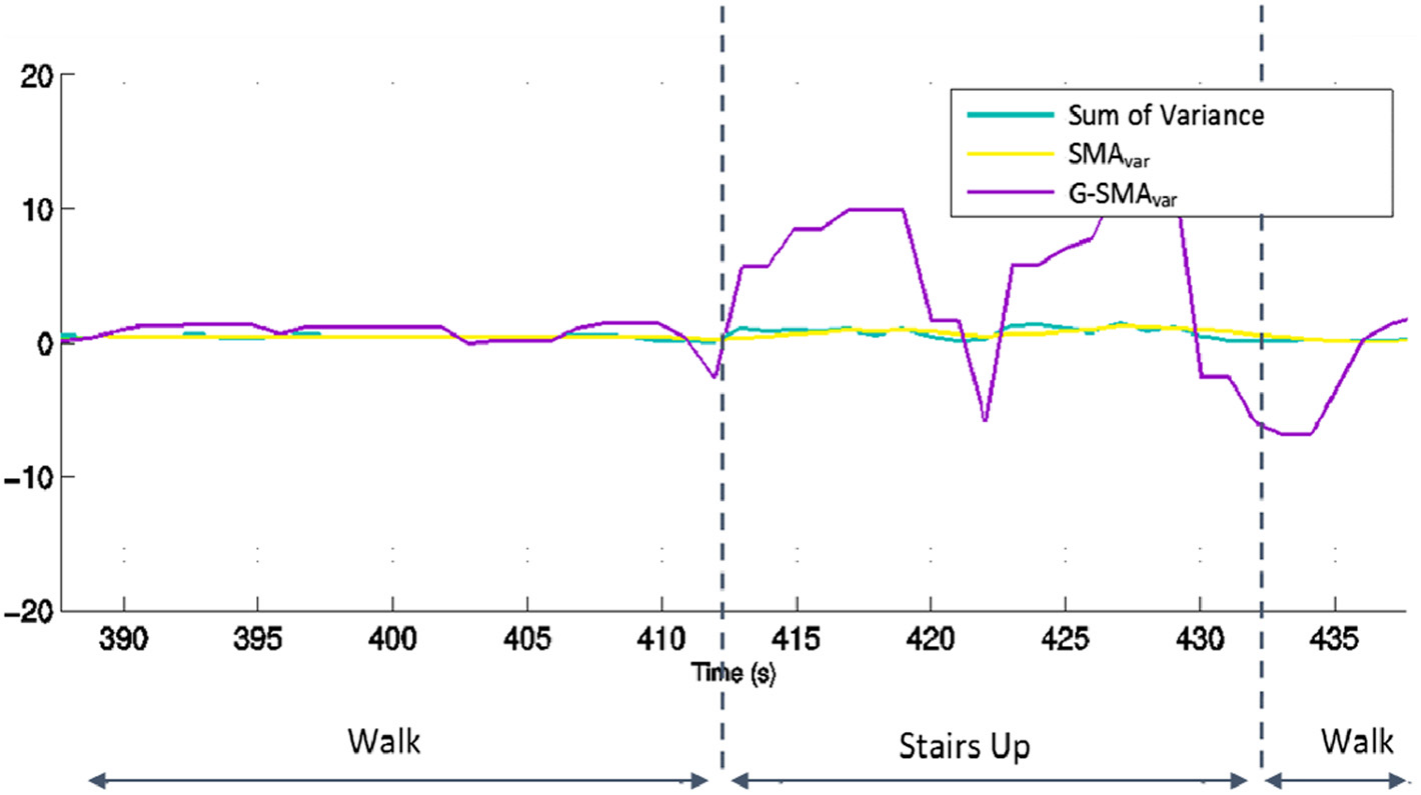
Plot of Sum of variance, SMAvar and G-SMAvar showing how these features change during waling and stair climbing activities

In stage three, the default classification was walking. If the participant walked for more than 5 s and the slope of G-SMAvar feature passed a threshold, then the activity was classified as climbing stairs. Figure 4 shows how G-SMAvar changes when a person is walking and when they are climbing stairs. The set of stairs used in this example had a landing in the middle, corresponding to the downward slope in G-SMAvar.

### Protocol

Data collection took place under realistic but controlled conditions. Participants follow a predefined path in The Ottawa Hospital Rehabilitation Centre or University Rehabilitation Institute, including living spaces within the rehab centres, and perform a consecutive series of mobility tasks: standing, walking, sitting, riding an elevator, brushing teeth, combing hair, washing hands, drying hands, setting dishes, filling the kettle with water, toasting bread, a simulated meal at a dining table, washing dishes, walking on stairs, lying on a bed, and walking outdoors [15] Appendix.

Before the trial, participant characteristics were recorded (i.e., age, gender, height, weight). Participants wore the smartphone in a holster attached to their right-front belt or pant waist, with the camera pointed forward. Trials were video recorded using a separate smartphone for activity timing comparison and contextual information. Video time was synchronized with the smartphone sensor output by shaking the phone at the beginning and end of the trial, providing a recognizable accelerometer signal and video event.

Gold-standard activity event times were manually identified from the video recordings. Each 1 s window was considered an occurrence. For example, sitting for 5 s was considered 5 occurrences. When segmenting the data, a 1 s window on either side of a change of state was considered part of the transition; to reduce error from inter-rater variability in identifying the start of an activity. Transitions were not considered when calculating outcomes. The number of 1 s instances (class distribution) of each activity is shown in Table 2. Since this is a realistic data sample representing activities of daily living, class imbalances occur. For example, there were more instances of walking or sitting than climbing stairs or lying down.

**Table 2:** Class distributions at each level

Activity	Able bodied	Stroke	Both
Stand	114 (27.1)	131 (37.0)	122 (32.9)
Sit	45 (6.6)	93 (26.9)	68.9 (30.8)
Lie	32 (7.0)	36 (4.3)	34 (6.0)
Walk	361 (32.9)	768 (239.4)	565 (266.5)
Upstairs	17 (2.5)	49 (25.9)	33 (24.4)
Small moves	95 (14.1)	135 (31.8)	115 (31.8)

Data analysis involved calculating the number of true positives (TP), true negatives (TN), false positives (FP) and false negatives (FN) in Matlab. Sensitivity, specificity, and F-scores were calculated for each individual, and the average and standard deviation of all participants were calculated for each activity. F-score was calculated as F = 2TP/(2TP + FP + FN). Results for each data window were compared to the gold-standard results from the video recording using descriptive statistics. Descriptive statistics and t-tests (*p* < 0.05) were used to compare sensitivity, specificity, and f-scores between able-bodied and stroke groups.

## Results

The WMMS performed similarly with able-bodied and stroke populations when detecting immobile and mobile states (stage 1), with all sensitivity and specificity results greater than 0.92 and F-scores greater than 0.94 (Table 3). No significant differences were found between groups for stage 1, although sensitivity and F-score for the stroke population were lower for immobile states and specificity was higher for mobile states.

**Table 3:** Average, standard deviation (in brackets), and differences between able-bodied and stroke groups for sensitivity, specificity, and F-score at stage 1

Activity		Sensitivity	Specificity	F-score
Stand	Stroke	0.920 (0.076)	0.997 (0.006)	0.944 (0.053)
	Able-bodied	0.963 (0.048)	0.997 (0.005)	0.975 (0.028)
	*p*-value	0.08	0.95	0.06
Large Moves	Stroke	0.997 (0.005)	0.920 (0.076)	0.994 (0.006)
	Able-bodied	0.997 (0.006)	0.963 (0.048)	0.993 (0.008)
	*p*-value	0.95	0.08	0.68

In stage 2, specificity and F-scores for stroke participants were significantly lower for stand detection, but specificity was greater than 0.94 for both groups (Table 4). Specificity for lie detection was significantly greater for stroke participants, but results for both groups were greater than 0.97. Sitting sensitivity and F-Score were lower than the other activities, with results for both groups less than 0.68.

**Table 4:** Average, standard deviation (in brackets), and differences between able-bodied and stroke groups for sensitivity, specificity, and F-score at stage 2

Activity		Sensitivity	Specificity	F-score
Stand	Stroke	0.826 (0.133)	0.940 (0.053)	0.701 (0.176)
	Able-bodied	0.903 (0.168)	0.987 (0.024)	0.917 (0.137)
	*p*-value	0.17	0.01	0.00
Sit	Stroke	0.533 (0.361)	0.987 (0.021)	0.568 (0.265)
	Able-bodied	0.646 (0.408)	0.983 (0.036)	0.673 (0.405)
	*p*-value	0.43	0.71	0.41
Lie	Stroke	0.794 (0.347)	1.000 (0.000)	0.824 (0.337)
	Able-bodied	0.943 (0.086)	0.979 (0.038)	0.871 (0.165)
	*p*-value	0.13	0.05	0.64
Walk	Stroke	0.997 (0.006)	0.961 (0.035)	0.993 (0.006)
	Able-bodied	0.997 (0.005)	0.966 (0.041)	0.989 (0.011)
	*p*-value	0.95	0.70	0.27

In stage 3, stand F-scores for stroke participants were significantly lower than the able-bodied group (Table 5). Lie specificity was significantly greater for stroke participants, but outcomes for both groups were greater than 0.98. For the stroke group, walk sensitivity and F-score were lower. Specificity was significantly lower for stair recognition and sensitivity and small movement recognition was poor for both groups.

**Table 5:** Average, standard deviation (in brackets), and differences between able-bodied and stroke groups for sensitivity, specificity, and F-score at stage 3

Activity		Sensitivity	Specificity	F-score
Stand	Stroke	0.759 (0.163)	0.883 (0.051)	0.512 (0.145)
	Able-bodied	0.878 (0.169)	0.886 (0.044)	0.728 (0.128)
	*p*-value	0.06	0.89	0.00
Sit	Stroke	0.533 (0.360)	0.978 (0.037)	0.529 (0.262)
	Able-bodied	0.646 (0.408)	0.975 (0.049)	0.660 (0.400)
	*p*-value	0.43	0.88	0.30
Lie	Stroke	0.794 (0.347)	1.000 (0.000)	0.824 (0.337)
	Able-bodied	0.943 (0.086)	0.982 (0.033)	0.871 (0.165)
	*p*-value	0.13	0.05	0.10
Walk	Stroke	0.514 (0.161)	0.903 (0.074)	0.646 (0.123)
	Able-bodied	0.643 (0.226)	0.932 (0.052)	0.734 (0.162)
	*p*-value	0.08	0.22	0.10
Stairs	Stroke	0.622 (0.260)	0.672 (0.107)	0.101 (0.085)
	Able-bodied	0.711 (0.384)	0.805 (0.123)	0.168 (0.142)
	*p*-value	0.46	0.00	0.13
Small Movements	Stroke	0.154 (0.156)	0.987 (0.016)	0.209 (0.179)
	Able-bodied	0.091 (0.102)	0.994 (0.01)	0.149 (0.163)
	*p*-value	0.20	0.23	0.35

## Discussion

This research demonstrated that a smartphone-based HAR approach can provide relevant information on human movement activities for both able-bodied and stroke populations, at a broad level of detail; however, sensitivity and specificity decrease as the classification tasks become more complex. Thus, our hypothesis that the WMMS would perform worse for stroke participants was valid at higher detail levels, but invalid at a broad classification level.

For stage 1, mobile and immobile activity states were well classified for both able-bodied and stroke populations. From the accelerometer-based HAR literature, activity classification accuracy ranged from 71 to 97 % [6, 16], with studies in the past two years typically reporting results from 92 to 96 % for able bodied [17, 18] and 82-95 % for older people [19]. Since this stage has only 2 classes, and the feature differences are large, thresholds can be set such that variability between people and populations has less of an effect on classification accuracy. Classification errors at stage1 may not be purely due to WMMS issues. For example, annotating gold-standard video can be difficult for small movements, such as washing dishes, since the person may move their body enough to be classified in a mobile state but human interpretation of the video could indicate an immobile state. The WMMS may provide a more consistent method of assessing an appropriate movement threshold for daily activity assessment since human raters could differ in their interpretation of movement-type and movement-onset during activities of daily living.

In stage 2, classification algorithm performance decreased when identifying if an immobile person was standing, sitting, or lying down. Specificity and F-score were significantly lower for stand detection and the algorithm performed poorly for sit identification, for both populations. Classification was based on static thresholds from a single feature (DifftoY). Since stroke can cause posture asymmetry during standing [20] and the stroke population was much older than the able-bodied sample, with posture changing with age [21], the DifftoY feature and threshold may not be sufficient to identify standing across populations, and could benefit from a combination of multiple features. In addition, inaccurate results could occur if the phone shifted or changed orientation during the trial. The therapist manually repositioned the phone during the trial for two stroke participants, one stroke participant unintentionally moved the smartphone with her paretic hand, and another participant intentionally re-adjusted his phone. The changed position may have affected application performance for activities that require a consistent phone orientation (i.e., standing, sitting, lying).

Inclination angle is typically used to classify posture when using a single accelerometer location [22]. In this case, sit identification relies on the pelvis tilting slightly back while sitting, which was not always the case in this study. For example, when a person sits at the dinner table they often lean forward to reach for objects or when eating. If the person did not sit back enough to pass the threshold before leaning forward, sitting was not identified. In many cases, stroke participants were detected as standing during the dinner table sequence. This reduced sitting sensitivity and standing specificity. Improvements in sit detection from one pelvis-worn sensor location could be achieved by using additional features or expanding the duration of sit analysis beyond the 1-s data window to compensate for forward-back transitions when sitting and performing daily activities (eating, office work, etc.). The DifftoY threshold setting was also attributed to classification problems for three of the able-bodied participants, for whom some sit periods were classified as lie. This outcome also demonstrated the importance of assessing HAR systems across a range of daily activities since the results would have been much better if only “pure” sit, stand, and lie tasks were included.

In stage 3, lower walk detection sensitivity and F-score were observed for the stroke group. The smartphone was worn on the right side of the pelvis and nine of the participants had right hemiparesis, thereby reducing pelvis movement on the right side and affecting sensor and feature output. In most cases, the people with right hemiparesis had slightly lower outcomes than those with left hemiparesis (<0.18 % difference in sensitivity and specificity), however the differences were not significant (*p* < 0.05). Many stroke participants wore the phone with cotton pants that had an elastic waist strap, which may have provided an inferior anchor point for the phone’s holster (i.e., as compared with a leather belt or fitted pants). This may have increased sensor signal variability for stroke participants. All able-bodied participants had a belt or more rigid pant waist. When used in practice, a viable HAR system must deal with mounting inconsistencies.

Stair specificity for the stroke group was significantly lower than the able-bodied group, and the algorithm performed poorly for stair recognition for all participants. F-score was low for both populations due to the high number of false positives detected, lowering the precision of classification. For five able-bodied people, the WMMS briefly detected “stairs” when lying down, then correctly re-identified the state as lie. This occurred because the feature used to detect stair climbing (covariance) increased during the stand-to-lie movement. Interestingly, this did not occur for stroke participants, perhaps due to a difference in bed height or a difference in mobility techniques when transitioning into a supine position. As with sitting, error correction over a longer duration would eliminate incorrect stair classification during the stand-to-lie transition. Stroke participants tended to rely more on the railing while climbing stairs. Multiple threshold settings for differing the stair ascent methods, or user-specific thresholds for stair identification, could be explored as a means of improving classification results. For example, one stroke participant ascended and descended the stairs in a step-by-step fashion that placed both feet on a single stair, thereby changing the sensor signals and hence affecting stair recognition. This is a common stair climbing strategy for the stroke population and persons with other mobility and walking limitations.

Small movements were not well classified for either population, resulting in a sensitivity of 0.09 for able-bodied participants and 0.15 for stroke participants. The small movements included in the trial (making toast, washing dishes, eating a meal etc.) did not always cause pelvis accelerations. Thus, accelerometer and gyroscope sensors located on the hip were not appropriate for detecting all activities. Other small movements, such as washing dishes or brushing teeth, caused the person to move their hips enough for the WMMS to classify a mobile state. The poor performance related to the difficulty in categorizing daily living human movements and difficulty setting small movement onsets when labeling gold-standard video. In future work, better methods are needed for gold file annotation, taking into account individual differences in how small movements are performed.

These results show that, while mobile and immobile classifications can be achieved with a relatively similar degree of accuracy for able-bodied and stroke participants, the WMMS had more difficulty with classification as the activity detail level increased, especially for the mobility affected stroke population. More research with pathological movement populations are required to understand how HAR algorithms need to be modified to accommodate for group and individual differences when performing activities of daily living.

Limitations in the current work include a moderate sample size from each population (15 people). The stroke group was not age matched to the able bodied group; therefore, age-related differences may have accounted for some differences in WMMS performance. However, the average ages for both groups were less than 60 years, which is not considered a senior population, thereby minimizing potential age effects. Stroke participants were in the sub-chronic phase and were capable of completing 30 min of walking. In the community, post-stroke populations may have lower mobility levels that could introduce greater movement variability, thereby decreasing WMMS performance. Since this study only used one smartphone model for testing. future work could evaluate algorithm performance with other smartphone based systems.

## Conclusions

In this paper, it was demonstrated that human activity recognition using a smartphone based system can be accomplished for both able-bodied and stroke populations. However, an increase in activity classification complexity leads to a decrease in WMMS performance with a stroke population. This validates the hypothesis that a HAR system developed using only able-bodied sensor data would perform worse when used to classify activities in a stroke population.

Sensor data and features produced by the different populations affected WMMS performance. The algorithm performed reasonably well for both stroke and able-bodied participants when differentiating between sit, stand, lie, and walk and between mobile and immobile states. When stair climbing and small movements were added to the classification, algorithm performance decreased. Additional features are recommended to more accurately identify sitting, standing, and lying, as well as stair identification, since stair signals are similar to level walking for many individuals. These features should be selected using data from people with differing mobility levels, so as not to over-fit the classifier to a young population with potentially more intense movements. The study results can be used to guide HAR system development for populations with differing movement characteristics.

## Appendix A: Activity Circuit

### The Ottawa Hospital Rehabilitation Centre, Ottawa, Ontario

Follow the participant and video their actions, on a second smartphone, while they perform the following actions, spoken by the investigator.

- From a standing position, shake the smartphone to indicate the start of the trial.
- Continue standing for at least 10 s. This standing phase can be used for phone orientation calibration.
- Walk to a nearby chair and sit down.
- Stand up and walk 60 m to an elevator.
- Stand and wait for the elevator and then walk into the elevator.
- Take the elevator to the second floor.
- Turn and walk into the home environment
- Walk into the bathroom and simulate brushing teeth.
- Simulate combing hair.
- Simulate washing hands.
- Dry hands using a towel.
- Walk to the kitchen.
- Take dishes from a rack and place them on the counter.
- Fill a kettle with water from the kitchen sink.
- Place the kettle on the stove element.
- Simulate placing bread in a toaster.
- Walk to the dining room.
- Sit at a dining room table.
- Simulate eating a meal at the table.
- Stand and walk back to the kitchen sink.
- Rinse off the dishes and place them in a rack.
- Walk from the kitchen back to the elevator.
- Stand and wait for the elevator and then walk into the elevator.
- Take the elevator to the first floor.
- Walk 50 m to a stairwell.
- Open the door and enter the stairwell.
- Walk up stairs (13 steps, around landing, 13 steps).
- Open the stairwell door into the hallway.
- Turn right and walk down the hall for 15 m.
- Turn around and walk 15 m back to the stairwell.
- Open the door and enter the stairwell.
- Walk down stairs (13 steps, around landing, 13 steps).
- Exit the stairwell and walk into a room.
- Lie on a bed.
- Get up and walk 10 m to a ramp.
- Walk up the ramp, turn around, then down the ramp (20 m).
- Continue walking into the hall and open the door to outside.
- Walk 100 m on the paved pathway.
- Turn around and walk back to the room.
- Walk into the room and stand at the starting point.
- Continue standing, and then shake the smartphone to indicate the end of trial.

### University Rehabilitation Institute, Ljubljana Slovenia

Follow the participant and video their actions, on a second smartphone, while they perform the following actions, spoken by the investigator.

- From a standing position, shake the smartphone to indicate the start of the trial.
- Walk down the hall to a chair in another room and sit down.
- Stand up and walk into the hall.
- Walk around the lobby and into the home environment
- Walk up to the sink and simulate brushing teeth.
- Simulate combing hair.
- Simulate washing hands.
- Dry hands using a towel.
- Walk to the kitchen.
- Take dishes from a table and place them on the counter.
- Fill a kettle with water from the kitchen sink.
- Place the kettle on the stove element.
- Simulate placing bread in a toaster.
- Walk to the dining room.
- Sit at a dining room table.
- Simulate eating a meal at the table.
- Stand and walk back to the kitchen sink.
- Rinse off the dishes and place them in a rack.
- Walk from the kitchen to the elevator.
- Stand and wait for the elevator and then walk into the elevator.
- Take the elevator to the third floor.
- Walk down the hall to a stairwell.
- Open the door and enter the stairwell.
- Walk up stairs (11 steps, around landing, 11 steps).
- Open the stairwell door into the hallway.
- Walk down the hall.
- Open the door and enter a room.
- Lie on a bed.
- Get up and walk to the door.
- Open the door and walk back down the hallway.
- Open the door and enter the stairwell.
- Walk down stairs (11 steps, around landing, 11 steps).
- Exit the stairwell and walk down the hallway back to the elevator.
- Stand and wait for the elevator and then walk into the elevator.
- Take the elevator to the first floor.
- Walk to the exit and walk outside.
- Walk through the parking lot to the underground parking entrance.
- Stand at the top of the ramp.
- Walk down the ramp, turn around, then walk up the ramp.
- Walk through the parking lot back to the entrance.
- Walk inside and back to the elevator.
- Stand and wait for the elevator and then walk into the elevator.
- Take the elevator to the second floor.
- Walk down the hall and stand at the starting point.
- Continue standing, and then shake the smartphone to indicate the end of trial.
